# A 35-Year Record (1987–2022) of Hg Concentrations in Two of the Fish Species Most Consumed by People Living in the Upper Madeira River Basin, Brazilian Amazon Region

**DOI:** 10.3390/toxics12020144

**Published:** 2024-02-10

**Authors:** Luiz Drude de Lacerda, Ronaldo de Almeida, Wanderley Rodrigues Bastos

**Affiliations:** 1 Laboratório de Biogeoquímica Costeira, Instituto de Ciências do Mar (LBC-LABOMAR), Universidade Federal do Ceará, Av. da Abolição, 3207 Meireles, Fortaleza 60165-081, CE, Brazil; 2 Programa de Pós-Graduação em Desenvolvimento Regional e Meio Ambiente, Laboratório de Biogeoquímica Ambiental Wolfgang C. Pfeiffer, Universidade Federal de Rondônia, Av. Pres. Dutra, 2967 Olaria, Porto Velho 76801-059, RO, Brazil; ronaldoalmeida@unir.br (R.d.A.); bastoswr@unir.br (W.R.B.)

**Keywords:** Amazon basin, Hg emissions, deforestation, ASGM, fish, total Hg

## Abstract

This study presents a 35-year record of total mercury (Hg) concentrations in the detritivore fish *Prochilodus nigricans* (Curimatã) and the carnivore *Cichla pleiozona* (Tucunaré), two of the most widely distributed, ecologically important and consumed fish species in the upper Madeira River Basin in the Western Brazilian Amazon. Fish samples from the major Madeira River and marginal lakes and tributaries were compared. Irrespective of site, Hg concentrations were higher in the carnivore fish compared to the detritivore. Hg concentrations increased 5-fold in *C. pleiozona* in the past three decades, whereas they remained relatively constant in *P. nigricans* when analyzing the entire 35-year period. When analyzed separately, fish in the main river and marginal lake and tributaries presented the same pattern of Hg variation, with a significant increase in Hg concentrations in the carnivore and in the detritivore in marginal lakes and tributaries but not in the main river. This was in line with the increase in methyl-Hg production in tributaries, mostly associated with deforestation in the past decade in the basin. Although an increase in direct emissions from artisanal gold mining also occurred in the past decade, this caused virtually no impact on fish Hg concentrations, suggesting atmospheric emission and deposition in forests and further export to water systems as an intermediate link with fish Hg concentrations.

## 1. Introduction

Emissions of mercury (Hg) in the Amazon region by artisanal and small-scale gold mining (ASGM) have been extensively studied in the literature. Early direct links between Hg emissions into rivers and fish contents, e.g., [1,2,3,4], equivocally suggested that all Hg released to rivers was bioavailable. However, subsequent speciation studies clearly demonstrated that most Hg released directly to rivers is elemental Hg^0^, with very low mobility and bioavailability [5,6,7]. These findings induced a gradual substitution of this simple model by a more elaborate one that includes changes in land use accompanied by emission and further oxidation of Hg^0^ to Hg^+2^ in the atmosphere, particularly during the dry season when the Amazon atmosphere is enriched with soot and ozone from forest burning, accelerating Hg^0^ oxidation and increasing deposition in soils and water bodies, followed by methylation and mobilization, e.g., [8,9,10,11,12]. 

Regardless of the main environmental pathway, human exposure to Hg in the region became among the highest in the world [13]. In addition, while in 2018 Brazil acceded to the Minamata Convention on Mercury (aiming to reduce anthropogenic Hg emissions and to improve public awareness of its health impacts) [14,15], policies and actions to reduce Hg use in ASGM have mostly been unsuccessful [16]. Indeed, a surge of gold mining has occurred in the Amazon in the past decade [17], and contamination of natural resources has again triggered public awareness and concern among the public and environmental authorities in Brazil.

High environmental exposure of humans to Hg is associated with fish consumption. People in traditional and indigenous riverside communities in the Amazon depend mostly on fish as a protein source. The estimated fish catch in Brazil is between 422,000 and 473,000 t yr^−1^, about 75% of which is in the Amazon region. The estimated regional per capita consumption rates range from 12.4 to 13.8 kg yr^−1^ for populations in the entire basin, and from 22.4 to 25.2 kg yr^−1^ for populations in floodplain areas [18]. In the upper Madeira River Basin, however, the per capita consumption of traditional subsistence communities is much higher, on the order of 140 kg yr^−1^ [19]. Fish consumption is the main pathway to human contamination and has been widely used throughout the Amazon Basin for biomonitoring environmental Hg. A recent article estimated that Brazil accounts for 21% of the global methyl-Hg load associated with fish consumption, which reaches about 1285 kg yr^−1^, including fish species caught in the mouth region of the Amazon River [20].

While short-term monitoring programs may be sufficient to determine source-impact association from simple point sources, long-term monitoring is necessary to assess changes in concentrations of pollutants, mostly when this derives from complex patterns, such as the case of Hg in the Amazon. In the present study, we report a 35-year record of Hg concentrations in two key fish species, the detritivore *Prochilodus nigricans* (Curimatá) and the carnivore *Cichla pleiozona* (Tucunaré). These are among the most widely distributed and consumed fish from the upper Madeira River Basin in the Western Amazon, aiming to associate Hg contamination with changes in Hg emissions due to changes in land use in the basin, the oldest and still most significant area of ASGM in the Western Amazon.

## 2. Materials and Methods

### 2.1. Study Area and Database

**Figure 1:**
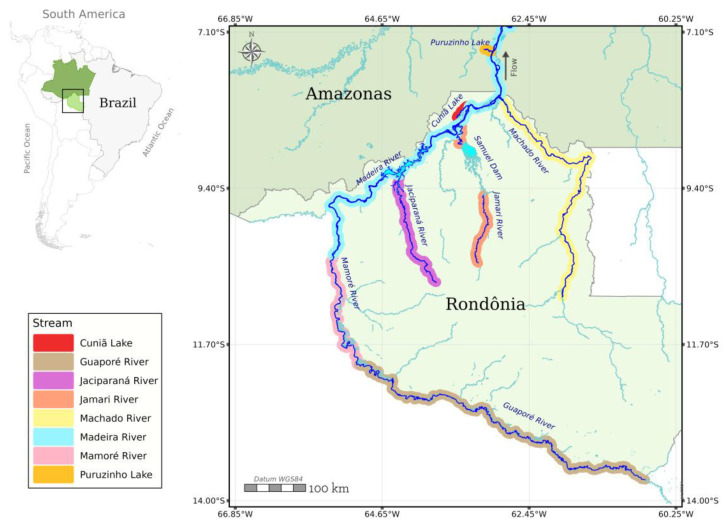
Study area showing the tributaries and lakes where the two fish species were collected in the Madeira River Basin, Western Amazon.

We analyzed the results from a database of Hg concentrations in fish available from the Wolfgang C. Pfeiffer Laboratory of Environmental Biogeochemistry of Rondônia Federal University, under the curation of W. R. Bastos. Two species with different foraging habits, the detritivore *Prochilodus nigricans* (Curimatã), *n* = 826, and the carnivore *Cichla pleiozona* (Tucunaré), *n* = 831, have available results dating back to the beginning of ASGM activity in the upper Madeira River Basin in the late 1980s (Figure 1). We selected results from individuals’ samples in the main river and its major tributaries (Jacy-Paraná, Jamari and Machado rivers) and from lakes (Cuniã and Puruzinho lakes) (Figure 1).

In the Madeira River Basin, commercial fishing for *P. nigricans* and *Cichla pleiozona* occurs mainly during the low-water period. *P. nigricans* (Characiformes: Prochilodontidae) is a species that undertakes reproductive migration from floodplains, lakes and small tributaries to the white-water rivers, such as the Madeira River, with well-oxygenated water. After reproduction, this species presents trophic migration, returning to the tributaries and lakes. This species has a wide distribution, also being found in the Amazon and Tocantins basins [21]. It basically feeds on organic debris and periphyton [22]. In turn, *C. pleiozona* (Perciformes: Cichlidae) is a species native to the Amazon River Basin with an opportunistic carnivorous feeding habit that enables it to dominate other species, including in artificial habitats such as reservoirs [23]. *Cichla pleiozona* is widely distributed in the upper Madeira River Basin, including the drainages of the Madre de Dios, Ben, Mamoré and Guaporé rivers [24]. The high-water transparency resulting from low suspended sediment transport favors visual predators such as *C. pleiozona*, an abundant species in this basin [25,26].

This study was approved by the relevant Brazilian government agency (SISBIO, registration numbers 58066-8, 17011-2 and 65585-1) for collection of biological material.

### 2.2. Sampling and Chemical Analysis

The field procedures and sampling, pretreatment of samples and analytical and quality control methods are detailed in previous publications [12,21] and briefly described below.

Over the years between 1987 to 2022, the fish samples were acquired by the Environmental Biogeochemistry/UNIR Laboratory team (Bastos, W.R. is a curator) from fishermen and local markets and identified to the species level. All individuals, independent of the sampling period, were treated similarly throughout the years. All have their biometry (size and weight) taken and an aliquot was taken from the muscle tissue of the dorsal region of each individual and transported under refrigeration and immediately frozen at −18 °C in the laboratory. The species have a median length of 24.0 cm (12–42 cm) and weight of 350 g (38–2100 g) for *P. nigricans* and 27.0 cm (14–50 cm) and 400 g (60–2700 g) for *C. pleiozona*. These size ranges that suggest the database included both adults and juvenile individuals. Sex was not recorded or taken into consideration in the analyses since previous work has demonstrated no difference in Hg concentrations between sexes in these two species [27]. 

For Hg determinations, approximately 500 mg of muscle tissue was weighed in duplicate (wet weight) after thawing at room temperature. After weighing, the samples were digested with solutions of 1:1 (*v*/*v*) H_2_SO_4_:HNO_3_ (Merck, Darmstadt, Germany) and 5% (*m*/*v*) KMnO_4_, (Merck, Darmstadt, Germany) for oxidation in a digester block at 70 °C for 1 hour. The following day, after adding drops of 12% (*m*/*v*), NH_2_OH.HCl (Merck, Darmstadt, Germany) to a final volume of 10.0 mL, Hg was determined by cold vapor-atomic absorption spectrophotometry (CV-AAS, FIMS-400, PerkinElmer, Waltham, MA, USA).

The analytical quality was controlled using reagent blanks in each sample batch, replicates of each sample, and certified reference material (CRM) acquired from the National Research Council Canada; dogfish muscle (DORM-2) and Tuna Fish (BCR-463) were acquired from the European Commission Joint Research Centre. The recovery percentage of these CRMs presented an average of 95% ± 12% throughout the period. 

### 2.3. Total Hg Emissions from ASGM and Deforestation

Deforestation rates were obtained from PRODES (Project to Monitor Deforestation in the Legal Amazon by Satellite)—the monitoring program of the Amazon Biome by the National Institute of Space Research [22] relative to the upper Madeira River Basin. PRODES has proven to be of great importance to plan public policies in the Amazon, and its accuracy indicates a level of precision close to 95%. Emissions of Hg from deforestation were estimated using emission factors calculated by different authors, including forest fires [28,29,30], degassing [31,32] and runoff following erosion [9,10]. An emission factor is the amount of Hg, emitted to the environment to produce a kg of gold, or from a ha of deforested area. The emission factors proposed by these authors were based on direct measurements of Hg vapor emissions from forest fires, different Hg fluxes from soils under different uses and modelling based on average concentrations of Hg in the forest biomass. These emission factors ranged from 2.7 to 7.8 g Hg ha^−1^ with an average of 5.9 g Hg ha^−1^. 

Production data from the region’s ASGM were obtained from annual reports of the mining sector [33,34], local prospector cooperatives [35] and the mining authorities of the Brazilian government and state of Rondônia [36,37]. Total Hg emissions were estimated using consistent and accredited emission factors (EFs) developed by Pfeiffer and Lacerda [5], Lacerda et al. [38] and UNEP [39]. In summary, the most precise estimations published by these authors were obtained from quantifying the amount of Hg and the respective gold production of about 740 mining operations in different areas in the Amazon region to provide an accredited average emission factor of 1.32 kg Hg per kg of gold produced.

### 2.4. Statistical Analyses 

Biometrical data were recorded to test for significant impacts of fish length and weight on Hg concentrations, since no significant relationship was found between biometric data and Hg concentrations (Figures S2 and S3, Supplementary Materials). We used the raw Hg concentrations directly without standardization of the results. All statistical calculations and graphing were performed using GraphPad Prism version 10.1.2 for Windows 10 (GraphPad Software, Boston, MA, USA). Biometric data and total mercury concentration in fish were tested for normality using the Shapiro–Wilk test. Since the data were not normally distributed, we used the nonparametric Kruskal–Wallis test to compare differences between groups, followed by Dunn’s post-hoc test for multiple comparisons. For all tests, a significance level of 5% was adopted (α = 0.05).

## 3. Results and Discussion

### 3.1. Mercury Emissions from ASGM and Deforestation

**Figure 2:**
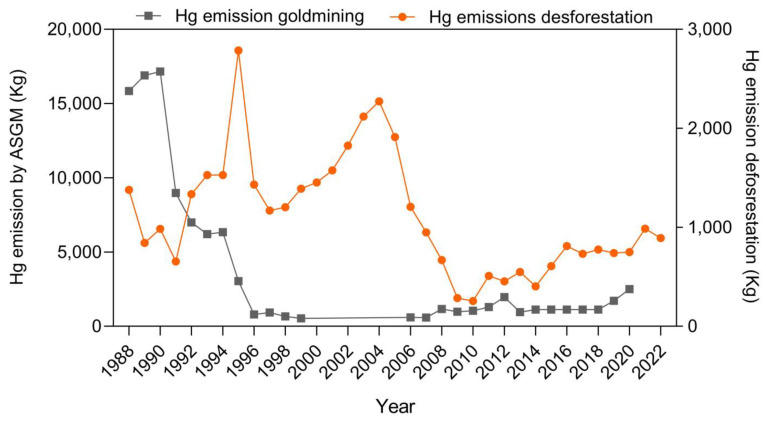
Estimated annual Hg emissions from deforestation and ASGM in the upper Madeira River Basin from 1988 to 2022. Emission factors [9,10,28,29,30,31,32] used in the calculations and deforestation rates [22] and ASGM [33,34,35,36,37,38,39] production are detailed in the Supplementary Materials.

The estimated emissions of Hg in the upper Madeira River Basin from deforestation and artisanal small scale gold mining (ASGM) from 1986 to 2022 are presented in Figure 2. Annual deforestation rates and gold production from ASGM are presented in the Supplementary Materials (Figure S1). 

Annual deforestation rates were higher from 1992 (2225 km^2^) to 2006 (2049 km^2^) and reached a maximum in 1995 of 4730 km^2^. These rates resulted in an estimated Hg emission varying from 1.20 to 2.79 tons per year. Starting in 2003, there was a steady decrease in deforestation rates, following strong federal enforcement of environmental regulations and policies, reaching a minimum of 684 km^2^ in 2014 and representing an estimated Hg emission of 0.40 ton. Unfortunately, from 2015 onwards, due to political and economic factors and weaking enforcement of environmental legislation, the deforestation rates increased again, reaching 1673 km^2^ in 2021 and a consequent estimated Hg emission of 0.99 tons. Deforestation was mostly driven by forest conversion into pasture and slash and burn agriculture releasing large amounts of Hg into the atmosphere. These activities occurred mostly along the margins of major tributary rivers. 

Annual gold production from ASGM in the upper Madeira River Basin reached a maximum in the late 1980s to the early 1990s, varying from 13 to 16 tons per year. Afterwards, gold production decreased to a minimum of 0.49 tons by 2006 but increased onwards to 1.9 tons in 2020. Estimated Hg emissions from ASGM peaked in 1990 at about 17.2 tons and remained high until 1994 (6.4 tons), decreasing thereafter and varying from 0.53 to 2.5 tons in 2020. 

Of notice is the construction of two hydroelectric dams that began operating in 2012 using surface water turbines on the upper Madeira River, but these resulted in relatively little flooding and had minimal little effects on Hg fish concentrations [12].

### 3.2. Long-Term Changes in Fish Mercury in the Madeira River Watershed

Total Hg concentrations were higher in the carnivore *C. pleiozona*, with a median concentration of 0.879 mg kg^−1^, varying from 0.010 to 3.10 mg kg^−1^. Lower median Hg concentrations were observed in the detritivore *P. nigricans* (0.158 mg kg^−1^), varying from 0.001 to 1.285 mg kg^−1^. Higher Hg concentrations in carnivores relative to detritivores have been frequently reported for Amazon fish [27]. 

Both fish species had non-significant correlations between weight and length versus total Hg concentrations, between length versus total Hg concentrations in *P. nigricans* (r_s_ = 0.0768, *p* = 0.0331, Supplementary Materials Figure S2) and weight vs. total Hg concentrations in *C. pleiozona* (r_s_ = 0.1396, *p* > 0.0001, Supplementary Materials Figure S3). These results were probably due to the large time span of the data, which includes different populations and age groups.

**Figure 3:**
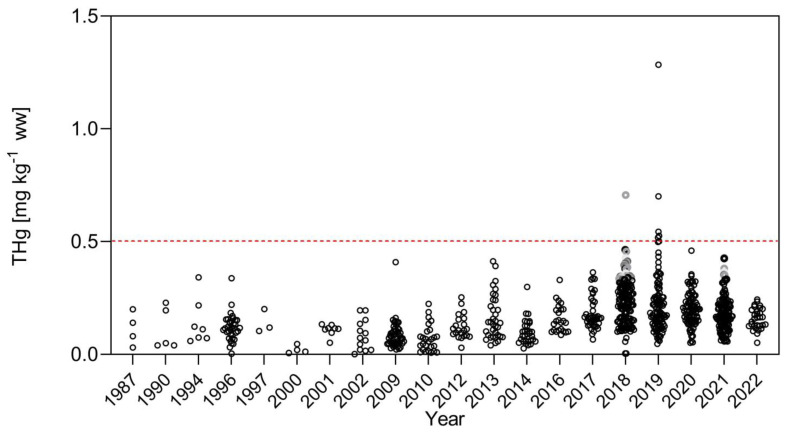
Temporal variation of Hg concentrations in *Prochilodus nigricans* (Curimatã, *p* < 0.0001, KW = 594.4, *n* = 826) in the upper Madeira River Basin (1987–2022), considering the entire dataset including main river, marginal lakes and tributaries. Dashed red line: limit of Brazilian legislation for non-carnivorous species.

**Figure 4:**
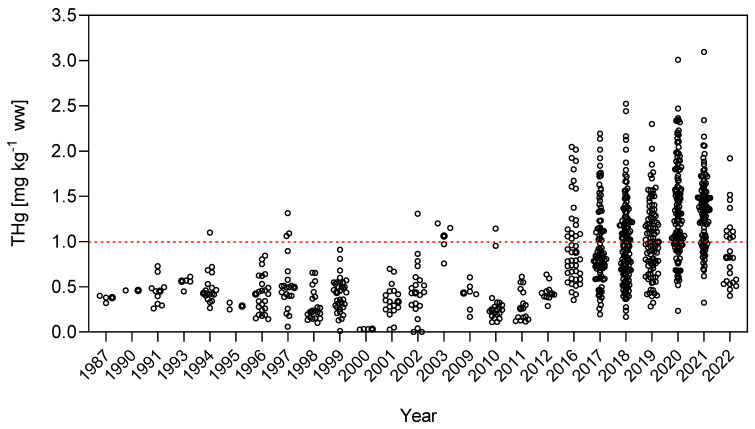
Temporal variation of Hg concentrations in *Cichla pleiozona* (Tucunaré, *p* < 0.0001, KW = 636.8, *n* = 831) in the upper Madeira River Basin (1987–2022) considering the entire dataset including main river, marginal lakes and tributaries. Dashed red line: limit of Brazilian legislation for carnivorous fish.

The nonparametric Kruskal–Wallis test was applied to compare the differences in the concentration of Hg in *C. pleiozona* and *P. nigricans;* when grouped by year, these showed that total Hg concentrations measured in the two fish species presented different temporal tendencies depending on their foraging habits. The detritivore *P. nigricans* showed no significant changes in Hg concentrations when using the entire database (KW = 594.4, *p* = n.s.) (Figure 3), although a slight increase in concentrations has occurred in the past decade. Total Hg concentrations in this species were always lower than the maximum legal limits of the Brazilian legislation for non-carnivorous fish (0.50 mg kg^−1^ ww) [40]. The Kruskal–Wallis test was also applied to compare the differences in Hg concentration in *C. pleiozona* with time. For this species, however, considering the entire database, there was a significant difference (KW = 636.8, *p* < 0.0001) in Hg concentrations. The carnivore *C. pleiozona* had a steady and significant increase in total Hg concentrations starting in 2010 (Figure 4). From then onwards, there has been a significant increase in the frequency of occurrence of concentrations higher than the legal limits of Brazilian legislation for carnivorous fish (of 1.00 mg kg^−1^) [40]. Between 2016 and 2022, total Hg concentrations peaked above 3.00 mg kg^−1^ ww, leading to average concentrations higher than the legal limits (Figure 4).

When using the entire dataset, we found no association between the variability of the Hg concentrations in the detritivore *P. nigricans* and the variability of ASGM activity and respective Hg emissions. There also was no association with land use changes (i.e., deforestation) (Figure 2). However, in the carnivore *C. pleiozona*, there was no clear association between the variability of ASGM and its Hg emissions. On the other hand, there was a consistent relationship between Hg concentrations in this carnivorous fish species and the variability of deforestation rates in the upper Madeira River Basin during the 35 years studied.

### 3.3. Temporal Trends in Hg Concentrations in Tributaries and Marginal Lakes versus Main River

Bastos et al. [12] compared Hg concentrations in fish between two periods (1990–2002 and 2009–2013) in the upper Madeira River Basin and found different trends in concentration variation according to diet. They reported significant increases in Hg concentrations between the two periods in the detritivore *Pterygoplicthys* sp. (cascudo), the planktivores *Hypophthalmus edentatus* (maraté-cameta) and *Hypophthalmus marginatus* (mapará), the omnivore *Triportheus albus* (sardinha) and the piscivore *Pinirampus pirinampu* (barba-chata). The exception was the generalist carnivore *Serrassalmus rhombeus* (piranha preta), which showed a significant decrease in Hg concentrations. Unfortunately, the authors did not report data on the two species reported here. They also observed a large variation between species of different trophic positions, sampling sites and feeding strategies in relation to total Hg concentrations, and the authors also showed a significant spatial variability depending on the type of aquatic system, main river versus marginal lakes and tributaries.

**Figure 5:**
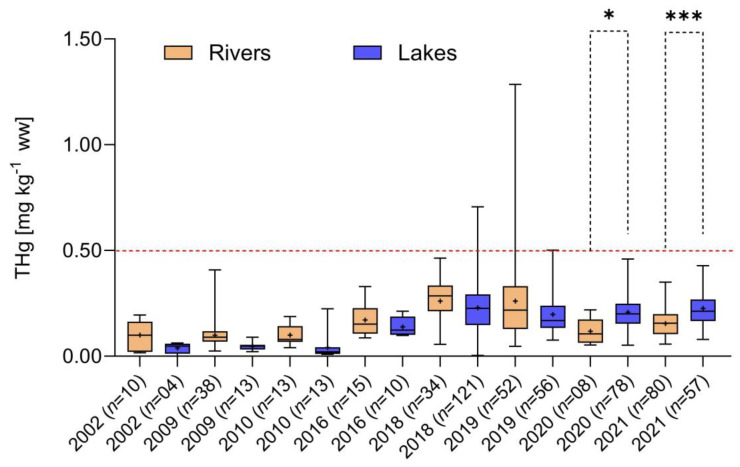
Temporal variation from 2002 to 2021 of total Hg concentrations in *Prochilodus nigricans* sampled in the main river, tributaries and marginal lakes, separately, of the upper Madeira River Basin (*** *p* < 0.0001, KW = 213.7, *n* = 602). Dashed red line: limit of Brazilian legislation for detritivores fish. The asterisks denote statistically significant differences at the 0.05 level (*).

**Figure 6:**
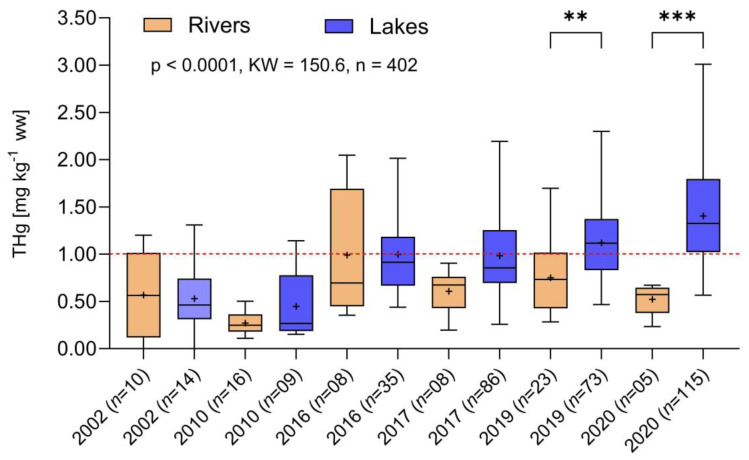
Temporal variation from 2002 to 2020 of total Hg concentrations in *Cichla pleiozona* sampled in the main river, tributaries and marginal lakes, separately, of the upper Madeira River Basin (*** *p* < 0.0001, KW = 150.6, *n* = 402). Dashed red line: limit of Brazilian legislation for carnivorous fish. The asterisks denote statistically significant differences at the 0.05 level (**).

Figure 5 and Figure 6 show total Hg concentrations in the two species according to their sampling site (main river versus tributaries and marginal lakes). In general, the different pattern of temporal variation in Hg concentrations was similar for the two species. The detritivore *P. nigricans* showed a significant increase in total Hg concentrations in the marginal lakes and associated tributaries, which was not observed in fish from the main river, especially in more recent years (Figure 5).

The carnivorous species *C. pleiozona* showed a significant increase starting in 2010 for fish in marginal lakes and tributaries (Figure 6), which was not observed in samples from the main river. However, contrary to the entire database when plotted together (Figure 4), there was no statistically significant increase observed in samples from the main river. Like the omnivores, a strong and significant increase in Hg concentrations was observed in the fish sampled in marginal lakes and tributaries. 

Dunn’s test was carried out for multiple comparisons, and both species had significantly higher Hg concentrations in tributaries/lakes compared to the main river in more recent periods (Figure 5 and Figure 6).

Environmental changes such as the spread of farming and stock breeding [10,41], deforestation for urban sprawl [6,42], and even establishment of landfills close to large cities [31,32] have been indicated as major processes promoting the remobilization of Hg from Amazon soils. These processes include degassing, erosion and increasing soil runoff, the formation of organic-Hg complexes and methylation. Degassing from soils and exposed sediments during the dry period [31,32], land use change, slash and burn agriculture [10,41] and deforestation by forest burning [42] are major drivers increasing the remobilization and availability of Hg from Amazon soils to aquatic environments. Seasonal flooding of marginal lakes [43] and inundation of forest areas [44,45], sometimes increased by river damming [46], are also suggested to augment Hg methylation and therefore methyl-Hg bioavailability to transport and incorporation in the aquatic biota. However, all these studies were the result of short-term monitoring and were in general spatially limited. Therefore, they fail to upscale the magnitude of the influence of those drivers on the bulk transport of Hg through Amazon rivers due to the lack of long-term monitoring of great extensions of Amazonian rivers and their tributaries. This study, for the first time, confirms the magnitude of the effect of land use drivers on Hg mobilization and eventual bioaccumulation in fish.

Different studies have highlighted the importance of flooded areas of tributaries and marginal lakes as major sources of methyl-Hg for the main rivers in the Amazon region [47] in the upper Madeira River basin [48]. Our results support those observations as suggested by the increasing total Hg content in fish, particularly from those waterlogged areas. These areas’ watersheds have experienced profound changes in land use, mostly deforestation for stock breeding and slash and burn agriculture since the beginning of the development plans for the state in the early 1980s, strongly promoted by the military governments. This trend increased the remobilization of highly bioavailable Hg species and their transport to major rivers. The impact of these dynamics on the fish Hg contamination is clearly identified through the long-term variability of Hg concentrations in fish presented in this study. 

Notwithstanding the recent sharp reduction in deforestation rates due to stronger enforcing of environmental legislation by the present Federal Government, other externalities may impact Hg remobilization from Amazon soils. Global climate change results in increasing temperature and extension and frequency of extended drought, strongly affecting the Amazon Forest. The expected reduction of forest cover will provoke more soil mobilization and consequently transport of highly bioavailable Hg water systems. Therefore, higher exposure to Hg is expected following these changes.

## 4. Conclusions

Fish Hg concentrations respond to different foraging habits and the land use changes in the upper Madeira River Basin, rather than the direct emissions from ASGM. Carnivorous species clearly showed significant increases in Hg concentration in the past decade, following intensification of deforestation in the basin. However, the detritivorous species also showed an increase in the same period, at least in marginal lakes and tributaries. Therefore, increasing Hg mobilization is a real and present threat caused by land use changes in the Amazon, specifically targeting traditional populations which strongly depend on fish as a major source of protein in their diet.

This study indicates that *Prochilodus nigricans* and *Cichla pleiozona* have potential as biomonitoring species of Hg levels in the Madeira River Basin. *Prochilodus nigricans* is an iliophagous detritivore species widely distributed in the Madeira River Basin. Therefore, it reflects the Hg accumulation in the nepheloid of bottom sediments. *Cichla pleiozona* is a piscivorous species and is considered a major predator throughout the Amazon Basin. *Cichla pleiozona* Hg concentrations reflect the incorporation of Hg in the aquatic trophic chain through the biomagnification process. Furthermore, *P. nigricans* (curimatã) and *C. pleiozona* (tucunaré) are important species in the diet of the local population and of great economic importance. Therefore, these species may be used to estimate the magnitude of the risk of human exposure, since the typically large ingestion of fish by local human populations is the main route of exposure to methyl-Hg in the upper Maderia River Basin as well as in the entire Amazon region.

Finally, externalities linked to global climate change, mostly increasing temperature and the frequency and intensity of drought events, may accelerate soil mobilization, and therefore the mobilization of highly reactive and bioavailable forms of Hg, resulting in increasing Hg content in the aquatic biota and exposure to Hg of the local, tradition population, still strongly dependent on fisheries.

## Data Availability

The data presented in this study are available upon request from the corresponding author.

## Supplementary Materials

The following supporting information can be downloaded at https://www.mdpi.com/article/10.3390/toxics12020144/s1. Figure S1: Estimated annual Hg emissions from deforestation and gold (Au) production by ASGM in the upper Madeira River Basin. EFs are from references listed in the bibliography and cited in the text. Emission factor is the amount of Hg emitted to the environment to produce a kg of gold or a ha of deforested area. Figure S2: Correlation between weight (red, r_s_ = −0.0391, *p* = 0.2625, *n* = 821), length (blue, r_s_ = 0.0768, *p* = 0.0331, *n* = 769) and total Hg concentrations in *Prochilodus nigricans* (Curimatá) from the upper Madeira River Basin. Figure S3: Correlation between weight (red, r_s_ = 0.1396, *p* < 0.0001, *n* = 831), length (blue, r_s_ = −0.0499, *p* = 0.1598, *n* = 796) and total Hg concentrations in *Cichla pleiozona* (Tucunaré), from the upper Madeira River Basin.
