# Supplementary material for: A 35-Year Record (1987–2022) of Hg Concentrations in Two of the Fish Species Most Consumed by People Living in the Upper Madeira River Basin, Brazilian Amazon Region
Source: Toxics. 2024 Feb 10;12(2):144. doi: 10.3390/toxics12020144 (PMC10892673; doi:10.3390/toxics12020144)
Supplement: Supplementary file 1 [file toxics-12-00144-s001.zip › toxics-2811902-supplementary.pdf]

Supplementary Materials: A 35-Year Record (1987– 2022) of Hg Concentrations in Two of the Fish Species Most Consumed by People Living in the Upper Madeira River Basin, Brazilian Amazon Region

Luiz Drude de Lacerda, Ronaldo de Almeida and Wanderley Rodrigues Bastos

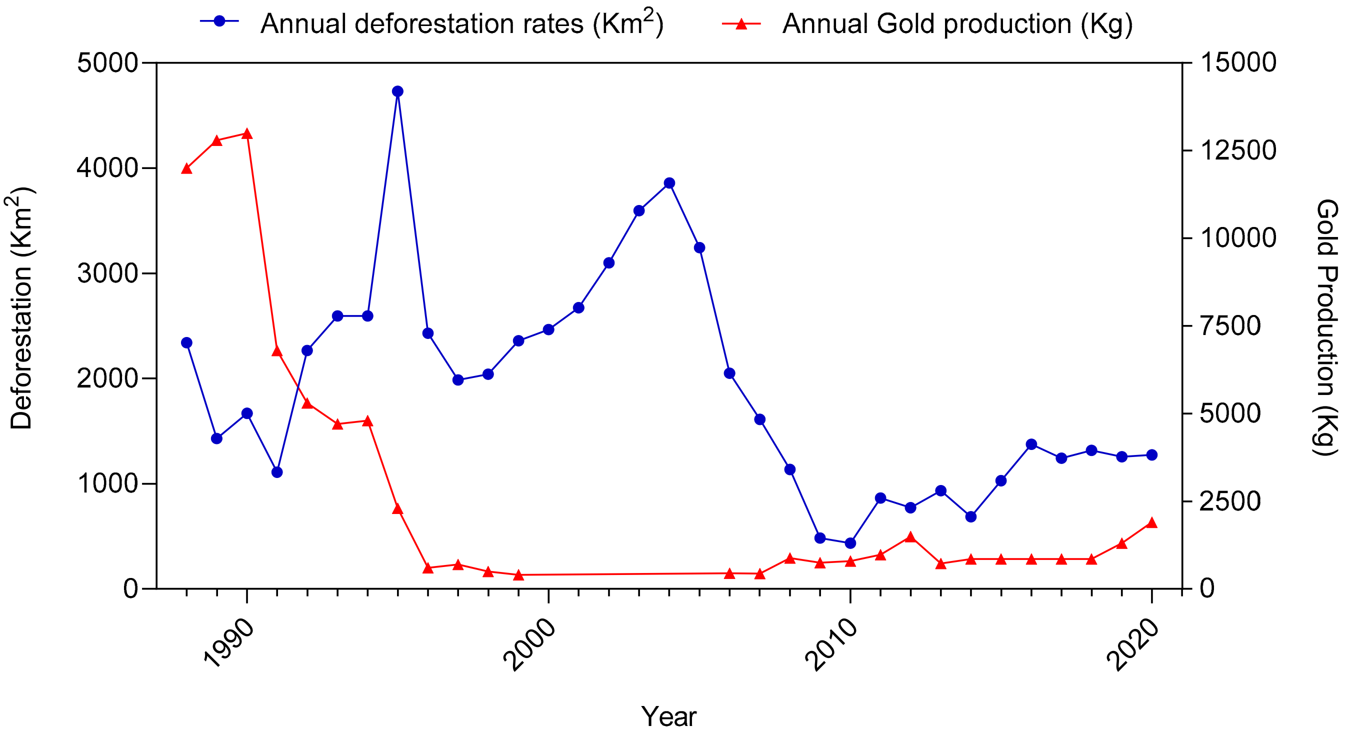

Figure S1. Estimated annual Hg emissions from deforestations and gold (Au) production by ASGM in the upper Madeira River Basin. EFs are from references listed in the bibliography and cited in text. Emission factor is the amount of Hg, emitted to the environment to produce a kg of gold, or a ha of deforested area.

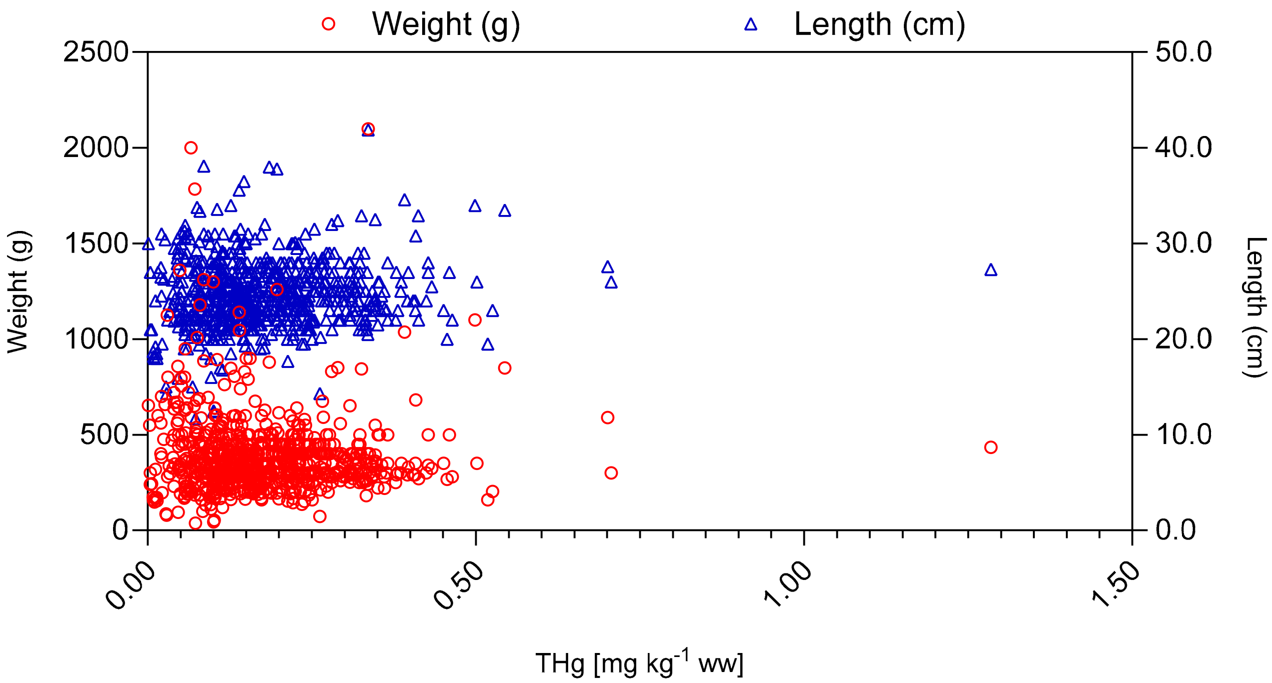

Figure S2. Correlation between weight (red, r_s = - 0.0391, p = 0.2625, *n* = 821), length (blue, r_s = 0.0768, p = 0.0331, *n* = 769) and total Hg concentrations in *Prochilodus nigricans* (Curimatá), from the Upper Madeira River basin.

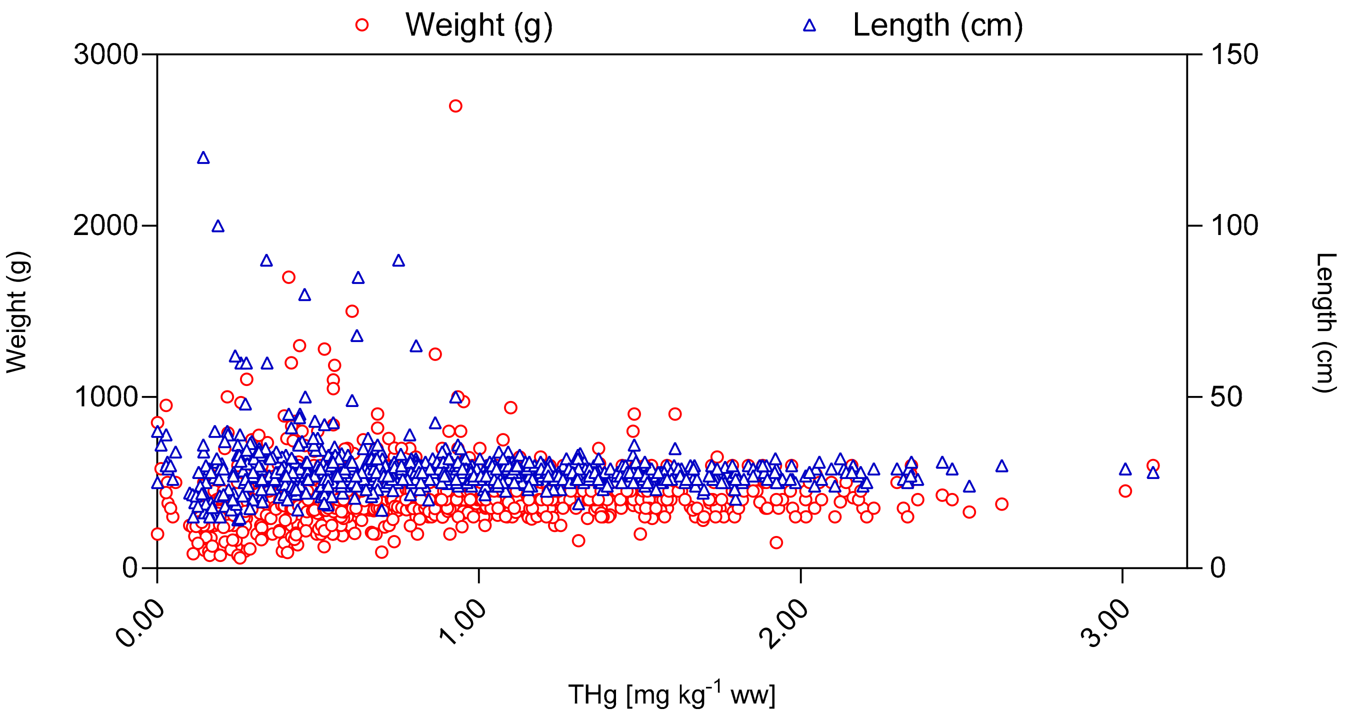

Figure S3. Correlation between weight (red, r_s = 0.1396, p < 0.0001, *n* = 831), length (blue, r_s = - 0.0499, p = 0.1598, *n* = 796) and total Hg concentrations in *Cichla pleiozona* (Tucunaré), from the Upper Madeira River basin.
